# Chromosome Preference During Homologous Recombination Repair of DNA Double-Strand Breaks in *Drosophila melanogaster*

**DOI:** 10.1534/g3.119.400607

**Published:** 2019-09-13

**Authors:** Joel Fernandez, Hanan Bloomer, Natalia Kellam, Jeannine R. LaRocque

**Affiliations:** Department of Human Science, Georgetown University Medical Center, Washington DC 20057

**Keywords:** DSB repair, homologous recombination, *Drosophila melanogaster*, interhomolog recombination, intrachromosomal recombination

## Abstract

DNA double-strand breaks (DSBs) are especially toxic DNA lesions that, if left unrepaired, can lead to wide-ranging genomic instability. Of the pathways available to repair DSBs, the most accurate is homologous recombination (HR), where a homologous sequence is used as a donor template to restore genetic information at the break site. While much of the biochemical aspects of HR repair have been characterized, how the repair machinery locates and discriminates between potential homologous donor templates throughout the genome remains elusive. We use *Drosophila melanogaster* to investigate whether there is a preference between intrachromosomal and interhomolog donor sequences in mitotically dividing cells. Our results demonstrate that, although interhomolog HR is possible and frequent if another donor template is not available, intrachromosomal donor templates are highly preferred. This is true even if the interhomolog donor template is less diverged than the intrachromosomal donor template. Thus, despite the stringent requirements for homology, the chromosomal location of the donor template plays a more significant role in donor template choice.

Genomic integrity is crucial for proper maintenance and dissemination of genetic information. However, cells frequently encounter DNA lesions that challenge the integrity of the genome. Of these lesions, double-strand breaks (DSBs) are especially toxic. They arise from both endogenous (*e.g.*, reactive metabolites, programmed DSBs in meiotic and immune cells) and exogenous sources (*e.g.*, ultraviolet or ionizing radiation, chemotherapeutic drugs) (reviewed in So *et al.* 2017). If left unrepaired, DSBs can lead to loss of genetic information, chromosomal rearrangements and apoptosis (Khanna and Jackson 2001; Hakem 2008). Consequently, inability to repair DSBs has been implicated in numerous genetic pathologies, including many cancers (reviewed in Jackson and Bartek 2009; Negrini *et al.* 2010; Janssen and Medema 2013).

Organisms have evolved multiple mechanisms to repair DSBs. These repair mechanisms have been broadly categorized into two pathways: non-homologous end joining (NHEJ) and homologous recombination (HR). NHEJ is active throughout the cell cycle and has been suggested to play a major role in DSB repair in mammalian cells (reviewed in Burma *et al.* 2006). In this repair pathway, the two broken ends of a DSB are directly ligated. Depending on the nature of the DSB, end processing including insertions and deletions (indels) may be required, resulting in potentially mutagenic repair outcomes. By contrast, HR uses a homologous sequence as a donor template to restore sequences at the broken ends of a DSB. Homologous donor templates may be located elsewhere on the chromosome or, in late S/G2 phases of the cell cycle, on the sister chromatid (collectively referred to as intrachromosomal HR) or on the homologous chromosome (interhomolog HR) in diploid organisms. HR most commonly occurs in S/G2 phases of the cell cycle in eukaryotic cells and can accurately repair DSBs with minimal or no loss of genetic information (reviewed in Shrivastav *et al.* 2008).

The molecular machinery, genetic components, and events required for HR repair have been well characterized (reviewed in San Filippo *et al.* 2008; Mazón *et al.* 2010; Heyer *et al.* 2010; Jasin and Rothstein 2013; Mimitou and Symington 2011; Bell and Kowalczykowski 2016). HR is initiated by resection of the 5′ end of a DSB, producing a single-stranded DNA (ssDNA) overhang. The 3′ overhang invades a homologous sequence that is used as a donor template for DNA repair synthesis. In mitotic cells, HR proceeds via synthesis-dependent strand annealing (SDSA), and the newly synthesized DNA strand dissociates from its homologous donor template. This strand then anneals to the other broken end of the DSB, restoring the intervening sequence through gene conversion, where the sequence that received the DSB is converted to the donor template sequence. While the biochemistry of HR repair is well characterized, the mechanism by which the HR repair machinery is able to locate and discriminate between potential homologous sequences across the genome (“homology search”) remains unclear.

An important factor in the homology search is the degree of homology between the DSB end and the homologous donor template. Several studies in eukaryotic systems have demonstrated that recombination between diverged sequences is highly suppressed; as the degree of homology between substrates decreases, the frequency of HR also decreases. In yeast, one to three mismatches in a stretch of homologous sequence is enough to suppress HR repair (Datta *et al.* 1996; Chen and Jinks-Robertson 1999). Suppression of repair between diverged sequences is also observed in homology-dependent template switching during break-induced repair (Anand *et al.* 2014). In mouse embryonic stem cells, a modest 1.2% sequence divergence results in sixfold reduction in HR (Elliott *et al.* 1998), a pattern consistent in human cells, though less pronounced (LaRocque and Jasin 2010). Recombination between diverged sequences is also markedly reduced in *Drosophila melanogaster*, with as little as 0.4% divergence significantly suppressing HR in a mismatch repair-dependent manner (Nassif and Engels 1993; Do *et al.* 2014; Do and LaRocque 2015).

In addition to homology, the location of the donor template relative to the DSB is also important. Numerous studies have found a pronounced usage of sister chromatid donor templates in both yeast and mammalian cells (Kadyk and Hartwell 1992; Liang *et al.* 1998; Johnson and Jasin 2000), though evidence exists for recombination between sequences on homologous, heterologous, or ectopic chromosomes (Lichten and Haber 1989; Malkova *et al.* 1996; Donoho *et al.* 1998; Richardson *et al.* 1998). Furthermore, intrachromosomal HR repair frequency in yeast varies according to the position of the donor template relative to the DSB: as the genomic distance between the DSB site and the homologous sequence increases, the frequency of HR decreases (Lichten and Haber 1989; Lee *et al.* 2016). This pattern is consistent in *Drosophila*, where HR repair of *P*-element-induced DSBs occurs more frequently between proximal sequences located on the same chromosome as the DSB (Engels *et al.* 1994), despite the observation that homologs are able to effectively serve as donor templates for HR repair (Rong and Golic 2003; Brunner *et al.* 2019). Taken together, these studies suggest that proximity plays a role in the efficiency and prevalence of HR repair and that the sister chromatid, being more proximal, is utilized more often as a donor template.

However, these studies lack in the ability to simultaneously track repair events from several homologous donor templates located throughout the genome. If given a choice, does the HR repair machinery preferentially distinguish between intrachromosomal donor templates and those located elsewhere in the genome? In the present study, we investigated this in mitotically dividing tissues of *Drosophila*, a strong model to test proximity effects given that homologous chromosomes have been shown to pair throughout the cell cycle (Metz 1916). We show that in the absence of an intrachromosomal donor template, interhomolog recombination is possible and occurs as frequently as intrachromosomal recombination. Yet, provided a choice between intrachromosomal and interhomolog donor templates, intrachromosomal donor templates are highly preferred. In accordance with previous studies, the degree of homology between the DSB and donor templates significantly affects the choice of donor template. Our findings suggest a sophisticated repair mechanism, whereby the HR repair machinery can distinguish between several donor templates while also discriminating against diverged sequences, regardless of genomic location.

## Methods

### DNA manipulations and cloning

All DNA construct development followed manufacturer’s protocol, unless otherwise stated. The DR-*white*Δ*9* construct (containing *Sce.white*, *yellow* transgene, *iwhite*Δ*9* donor template, and attB sequence) was created by a two-step cloning process. First, the *iwhite*Δ*9* donor template was created by identifying a spontaneous HR repair event from the original DR-*white* assay (Do *et al.* 2014) that resulted in orange-colored eyes. The repair event contained a 9-bp deletion at the original *Sac*I site in the *white* cDNA, resulting in a 3-amino acid deletion and reduced *w+* gene product (thus orange eyes). This repair event was used as a template for PCR amplification of *iwhite*Δ*9* fragment using CloneAmp HiFi PCR Pre-mix (Clontech) with primers 5′ GCTCCACCGCGGTGGCGGCCGCTTGGCCAAGAGGATCAGGAGCTA (forward, contains underlined *Not*I sequence) and 5′ CTTGATATCGAATTCCTGCAGTTGCAGATCGGCGGCGGAGAAGTTAA (reverse, contains underlined *Pst*I sequence). The *iwhite*Δ*9* PCR fragment with flanking restriction sites was cloned into *Not*I/*Pst*I–digested pBlueScript.KS^−^.attB vector (pBSKS^–^.attB; described previously in Do *et al.* 2014) using In-Fusion Cloning (Clontech) to create pBSKS^−^.*iwhite*Δ*9*.attB. Next, a *Sce.white* and *yellow+* fragment from the original DR-*white* construct (Do *et al.* 2014) was amplified with primers 5′ GAGCTCCACCGCGGTGGCGGCCGCCAAGTTTGTACAAAAAAGCAG (forward, contains underlined *Not*I sequence) and 5′ GCTCCTGATCCTCTTGGCCAAGCGGCCGCCAACTTTATTATACAAAGTTGTTT (reverse, contains underlined *Not*I sequence) using HiFi PCR Pre-mix. The *Sce.white_y*+ PCR fragment with flanking restriction sites was cloned into *Not*I–digested pBSKS^−^.*iwhite*Δ*9*.attB vector using In-Fusion Cloning to create DR-*white*Δ*9*. Similarly, for the *Sce.white* DSB recipient construct (containing *Sce.white*, *yellow* transgene, and attB sequence), the *Sce.white_y+* PCR fragment with flanking *Not*I restriction sites was cloned into *Not*I–digested pBSKS^−^.attB vector using In-Fusion Cloning.

For *iwhite* interhomolog donor construct (containing *yellow* transgene, *iwhite* donor template, and attB sequence), the *y+* transgene was amplified from the original DR-*white* construct with primers 5′ GAGCTCCACCGCGGTGGCGGCCGCCAACTTTTCTATACAAAGTTG (forward, contains underlined *Not*I sequence) and 5′ GCTCCTGATCCTCTTGGCCAAGCGGCCGCCAACTTTATTATACAAAGTTGTTT (reverse, contains underlined *Not*I sequence) using HiFi PCR Pre-mix. The *y*+ PCR fragment with flanking *Not*I restriction sites was cloned into *Not*I-digested pBSKS^-^.*iwhite*.attB vector using In-Fusion Cloning to create *iwhite* interhomolog donor construct. The *iwhite*Δ*9* interhomolog donor construct (containing *yellow* transgene, *iwhite*Δ*9* donor template, and attB sequence) was similarly created by inserting the *y*+ PCR fragment with flanking *Not*I restriction sites into *Not*I–digested pBSKS^−^.*iwhite*Δ*9*.attB vector using In-Fusion Cloning.

### Drosophila stocks and genetics

*Drosophila* were maintained on standard NutriFly Bloomington Formulation medium (Genesee Scientific, San Diego, CA) at 25° with 12-hour light/dark cycles.

The *white*^Δ^ allele was created using CRISPR/Cas9 tools. Two sequences homologous to the start (5′ GTGTGAAAAATCCCGGCAAT) and stop (5′ ACATATATCCGAAATAACTGCC) codon regions of the *white* gene were cloned adjacent to guide RNA (gRNA) scaffolds in the pCFD4 *Drosophila* expression vector as described in (Port *et al.* 2014) and CRISPR Fly Design (http://www.crisprflydesign.org). Briefly, a pCFD4 fragment including the two described sequences were amplified from pCFD4 (Addgene) with primers 5′ TATATAGGAAAGATATCCGGGTGAACTTCGTGTGAAAAATCCCGGCAATGTTTTAGAGCTAGAAATAGCAAG (forward) and 5′ ATTTTAACTTGCTATTTCTAGCTCTAAAACACATATATCCGAAATAACTGCCGACGTTAAATTGAAAATAGGTC (reverse) using HiFi PCR Pre-mix. The amplified PCR fragment was inserted into *Bbs*I-linearized pCFD4 vector using In-Fusion Cloning. Positive clones, identified by restriction digest, were sequenced (5′ GACACAGCGCGTACGTCCTTCG) to confirm accurate incorporation of gRNA sequences. pCFD4 vector with incorporated gRNAs were injected into NIG-FLY CAS-0001 embryos (genotype *y^2^ cho^2^ v^1^*; attP40{*nos-Cas9*}/*CyO*) (BestGene). G0 males were isolated and crossed into balancer lines to remove the *Cas9* transgene. G1 progeny were phenotypically screened for white eyes, crossed out to balancer lines, and their DNA extracted using the previously described DNA Preparation Protocol (Gloor *et al.* 1993). Briefly, genomic DNA was isolated using Squishing Buffer (50 µL; 10 mM Tris-Cl pH 8.2, 25 mM NaCl) and Proteinase K (10 µg), incubated at 37° for 30 min, followed by 95° inactivation for five minutes. PCR was performed using SapphireAmp Fast PCR Master Mix (Clontech) with primers 5′ GACAGCGAAAGAGCAACTACG (forward) and 5′ ACCAGGTTCTTTCGATTACCTC (reverse) flanking the white coding exons. Successful mutant lines were sequenced (5′ GACAGCGAAAGAGCAACTACG) to confirm deletion of the endogenous *white* sequence.

For recombination assays, purified DR-*white*Δ*9*, *Sce.white*, *iwhite*, and *iwhite*Δ*9* constructs were injected and integrated at the 51C1 locus of FlyC31 line M{3xP3-RFP’}ZH-51C using the PhiC3 integrase system (Bischof *et al.* 2007) (BestGene). Stable transformants were selected based on *y*+ expression and locus integration confirmed by PCR using primers 5′ CTGCAACTGCAGGAATTCG (forward) and 5′ GTCGTCCAGGCCTCGTTAAT (reverse). One line of each was selected based on fertility and health for downstream applications.

The heat-inducible *I-SceI* transgene is located on Chromosome *2* and linked to the dominant marker Sco (*Sco*^–^) (Rong and Golic 2003). For interhomolog recombination assays, this line was used to establish a recombinant line of *I-SceI* transgene and *iwhite* or *iwhite*Δ*9* constructs using standard genetic techniques. Briefly, crosses were set up for recombination to occur between the *I-SceI* transgene and the *iwhite* or *iwhite*Δ*9* constructs on Chromosome *2* within the female germline. Potential recombinant events in the next generation expressing *y*+ transgene and exhibiting *Sco*^–^ phenotype were isolated. To confirm recombination, genomic DNA of potential recombinants was isolated using Squishing Buffer and PCR performed using SapphireAmp Fast PCR Master Mix with *I-SceI*-specific primers 5′ CCAGCTGATCGAACTGAACA (forward) and 5′ CGCAGACCCTTAACCAGGTA (reverse).

### DSB repair assays

DSB repair assays were performed based on protocol described previously (Do *et al.* 2014). To induce DSBs, females containing the DSB recipient chromosome (either *Sce.white*, DR-*white* or DR-*whiteΔ9*) were crossed to males containing the heat-inducible *I-SceI* transgene (Rong and Golic 2003). For intrachromosomal repair assays, females carrying DR-*white* or DR-*white*Δ*9* were crossed to males containing the *I-SceI* transgene alone. For interhomolog repair assay, females carrying the *Sce.white* construct were crossed to males containing *I-SceI* transgene and either *iwhite* or *iwhite*Δ*9*. For intrachromosomal/interhomolog choice assay, females carrying DR-*white* were crossed to males containing *I-SceI* and *iwhite*Δ*9*, while females carrying DR-*white*Δ*9* were crossed to males containing *I-SceI* and *iwhite*. After three days, flies were removed and zero- to three-day old embryos and larvae were heat-shocked in a 38° water bath for one hour. Single F1 males containing both the DSB recipient chromosome (*Sce.white*, DR-*white* or DR-*white*Δ*9*) and the heat-inducible *I-SceI* transgene (along with the corresponding *iwhite*Δ*9* or *iwhite* sequence) were crossed to four–five *y w* tester females in vials. For each experiment/condition, vials containing ≥ 20 F2 progeny from 63-80 individual male germlines were scored. HR repair frequency was determined in F2 progeny containing *Sce.white*, DR-*white*, or DR-*white*Δ*9*.

### Molecular analyses of DSB repair events using TIDE

Genomic DNA was extracted from whole fly samples using a genomic DNA prep protocol adapted from Sullivan, Ashburner, and Hawley (2000). Single, adult flies were homogenized in Buffer A (50 μL; 100 mM Tris-Cl pH 7.5, 100 mM EDTA, 100 mM sodium chloride, 0.5% SDS) and incubated at 65° for 30 min. Buffer B (100 μL; 1.4 M potassium acetate, 4.3 M lithium chloride) was added and the mixture incubated on ice for 30 min. Samples were centrifuged at 13,200 rpm for 15 min at 4°. Supernatant was transferred to tubes containing 100 μL of isopropanol and centrifuged at 13,200 rpm for 10 min at room temperature. The DNA pellet was washed with 250 μL of cold 70% ethanol, air-dried and resuspended in 20 μL H_2_O. PCR reactions were performed on 100 ng of purified genomic DNA using SapphireAmp Fast PCR Master Mix. The target I-*Sce*I recognition site in *Sce*.*white* was amplified using primers 5′ GTGGATCAGGTAATCCAGG (forward) and 5′ CTTAAGCCATCGTCAGTTGC (reverse) under the following conditions: three minutes at 94°; 30 s at 94°, 30 s touchdown at 66° (-0.5°/cycle) and 30 s at 72° (16x); 30 s at 94°, 30 s at 58°, 30 s at 72° (20x); five minutes at 72° (1x); and held at 12°. PCR products were purified using the Wizard SV Gel and PCR Clean-Up System (Promega) and sequenced (5′ GAGCCCACCTCCGGACTGGAC). Sequences (Supplemental Files S2-26) were analyzed using the TIDE (Tracking across Indels by DEcomposition) algorithm, a computational protocol previously described (Brinkman *et al.* 2014) and customized for the DR-*white* assay (Janssen *et al.* 2016). The algorithm was further modified to include indels of up to 35 nucleotides surrounding the I-*Sce*I DSB site in the *Sce.white* sequence (Supplemental File S1). HR products were identified as conversions of the I-*Sce*I sequence to either the wild-type *white*+ sequence (a 23-bp deletion) or the modified *iwhite*Δ*9*+ sequence (a 32-bp deletion). Other insertions and deletions of up to 35 nucleotides were categorized as NHEJ with processing products. An output of 0 nucleotide indels was classified as the control I-*Sce*I recognition sequence where neither HR nor NHEJ with processing repair events occurred.

### Data Availability

Strains and plasmids are available upon request. Supplemental Table S1, the R script algorithm (Supplemental File S1), and sequence files (Supplemental Files S2-S26) used for TIDE analyses are available on FigShare. https://doi.org/10.25387/g3.9271007.

## Results

### Homologous recombination is suppressed in DR-whiteΔ9

**Figure 1:**
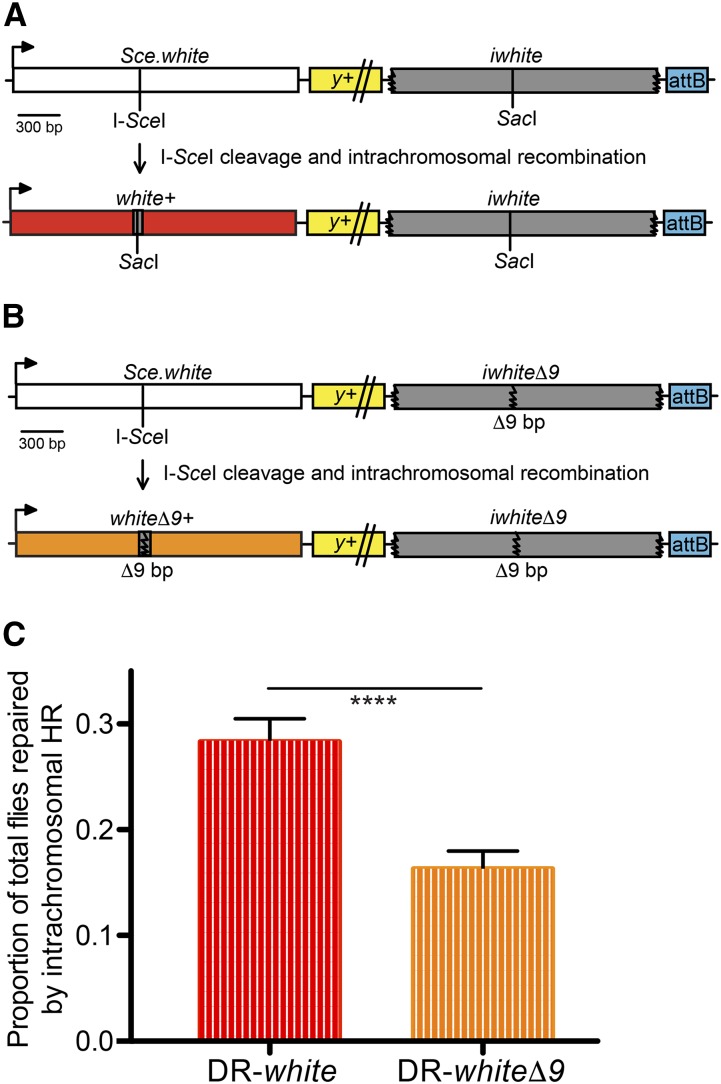
Sequence divergence significantly affects the frequency of homologous recombination repair. (A) The DR-*white* assay measures the frequency of intrachromosomal HR repair. An I-*Sce*I recognition sequence is inserted into the wild-type *Sac*I recognition site of *white* cDNA, resulting in a defective *white* sequence (*Sce.white*; white box). The downstream *white* sequence is defective because of 5′ and 3′ truncations (*iwhite*; gray box). Integration of DR-*white* is targeted using the attB sequence and followed with the *yellow* (*y*+) transgene (yellow box; not to scale). HR repair of an I-*Sce*I-induced DSB results in gene conversion of the *Sce.white* sequence to *white*+ (red box), resulting in red-eyed progeny. (B) The DR-*white*Δ*9* assay replaces the downstream *iwhite* sequence with *iwhite*Δ*9*, which contains a 9-bp deletion including the wild-type *Sac*I site in addition to 5′ and 3′ truncations (gray box). HR repair results in gene conversion of the *Sce.white* sequence to *white*Δ*9*+ (orange box), resulting in orange-eyed progeny. (C) F2 progeny for 38 individual male germlines of DR-*white* and 40 of DR-*white*Δ*9* were scored for eye color. The average intrachromosomal HR frequency out of total flies scored are shown. Error bars are S.E.M.; ****P < 10^−4^ (two-tailed unpaired Student’s *t*-test).

The previously characterized DR-*white* assay effectively measures intrachromosomal HR repair frequency in the *Drosophila* male premeiotic germline (Figure 1A)(Do *et al.* 2014; Do and LaRocque 2015; Janssen *et al.* 2016; Delabaere *et al.* 2017; Ertl *et al.* 2017). Briefly, flies containing the DR-*white* reporter assay and the *I-SceI* transgene are heat shocked to induce *I-SceI* expression in all cells, resulting in DSB formation followed by repair. Premeiotic germline events are isolated by crossing individual males to tester females and scoring F2 progeny for eye color (red eyes indicate gene conversion to wild-type *white* sequence).

Discriminating between interhomolog and intrachromosomal HR requires a distinguishing allele on the homolog that can reliably detect a choice between intrachromosomal and interhomolog repair. A novel *iwhite*Δ*9* sequence, containing a 9-bp deletion at the wild-type *Sac*I site, was identified that can serve as an HR donor template and results in a distinguishing phenotype of orange eyes. However, the sequence divergence between the DSB recipient and the new *iwhite*Δ*9* donor template could potentially confound measures of interhomolog HR repair frequency. Thus, two modifications were made to the existing DR-*white* assay to establish if homology differences would affect HR repair frequency in our assay.

First, to ensure that the endogenous *white* gene on Chromosome *X* could not serve as a donor template for repair and distort measures of HR frequency, the *white* coding region was deleted via CRISPR-Cas9 methods. The *white*^Δ^ allele contains a complete deletion of the *white* coding exons and intervening introns and a 7-bp insertion at the end-joining repair junction (5′ CTTGTTA). The novel *white*^Δ^ mutant allows experimental control over the donor templates available for repair. Thus, all genetic crosses described herein were carried out in a *white*^Δ^ mutant background.

Second, the DR-*white* assay was modified to create the DR-*white*Δ*9* assay with similar features: S*ce.white*, *yellow* transgene, and an attB targeting sequence (Figure 1B)(Do *et al.* 2014). Unique to previous studies, DR-*white*Δ*9* exchanges the downstream *iwhite* donor template for the *iwhite*Δ*9* donor template. In a *white*^Δ^ mutant background, the downstream *iwhite*Δ*9* provides the only homologous donor template for repair. Repair of I-*Sce*I-induced DSBs via intrachromosomal HR in DR-*white*Δ*9* results in gene conversion of *Sce.white* to a *white*Δ*9*+ sequence producing orange eyes in the F2 generation (Figure 1B).

To determine whether the deletion in *iwhite*Δ*9* affects HR repair frequency, we compared the frequency of intrachromosomal HR in both DR-*white* and DR-*white*Δ*9*. Recombination in DR-*white*Δ*9* decreased roughly 40% as compared to the original DR-*white* (16.3 ± 1.7% and 28.3 ± 2.2%, respectively; *P* < 10^−4^, two-tailed unpaired Student’s *t*-test; Figure 1C). While unsurprising, this finding revealed that the two repair donor templates, *iwhite* and *iwhite*Δ*9*, are not equivalent in terms of repair frequency. Therefore, each construct was tested separately to mitigate any confounding effects of sequence divergence.

### Interhomolog recombination is frequent in the male premeiotic germline

**Figure 2:**
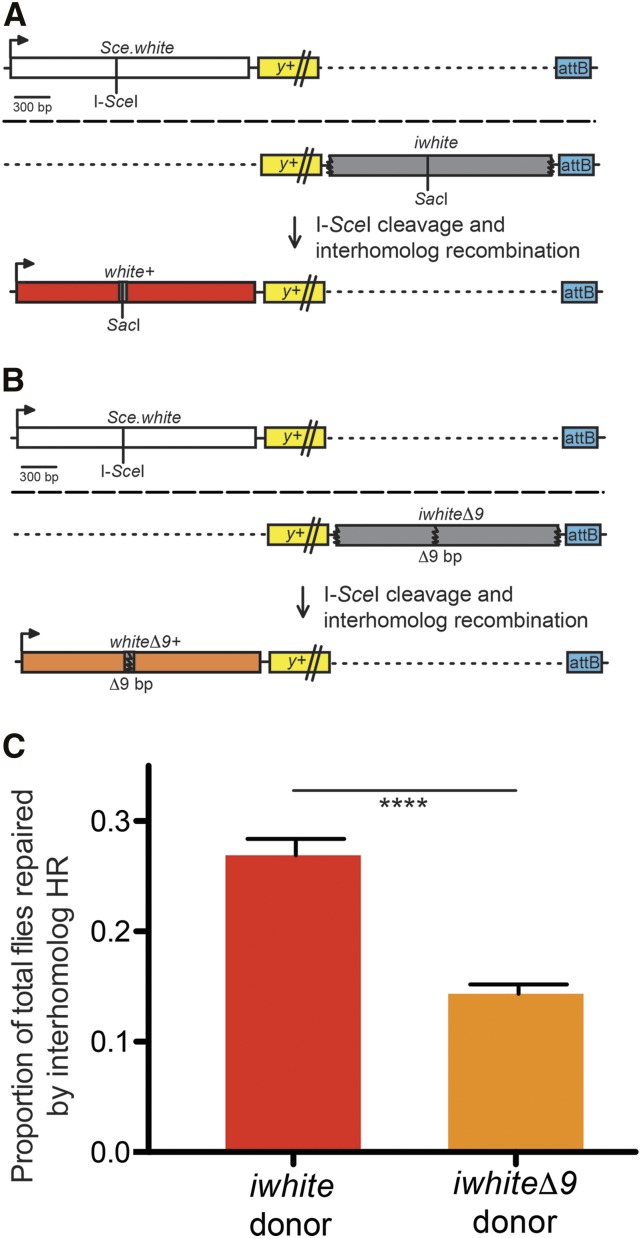
Interhomolog HR repair is possible and as frequent as intrachromosomal HR repair. (A) The interhomolog repair assay targets the *Sce.white* construct (white box) to Chromosome *2* and an *iwhite* donor template on the same allelic locus (gray box; homologs are separated by long dashed line). HR repair of an I-*Sce*I induced DSB from the homolog results in gene conversion of the *Sce.white* sequence to *white*+ (red box), resulting in red-eyed progeny. Graphics are aligned with the *y*+ transgene and attB sequence in order to compare with other figures. Dotted lines are for alignment purposes only. (B) The same assay is performed using the *iwhite*Δ*9* donor template (gray box), instead of *iwhite*, on the allelic Chromosome *2*. HR repair results in gene conversion of the *Sce.white* sequence to *white*Δ*9*+ (orange box), resulting in orange-eyed progeny. (C) F2 progeny for 65 individual male germlines with *iwhite* interhomolog donor template and 80 with *iwhite*Δ*9* interhomolog donor template were scored. The average interhomolog HR frequency out of total flies scored are shown. Error bars are S.E.M.; ****P < 10^−10^ (two-tailed unpaired Student’s *t*-test).

To confirm that interhomolog recombination is possible and detectable in our system, the interhomolog repair construct *Sce.white*, without a downstream intrachromosomal donor template, was utilized. Females carrying *Sce.white* were crossed with males carrying the *iwhite* or *iwhite*Δ*9* sequence on the allelic locus (Figures 2A and 2B). Gene conversion of the I-*Sce*I-induced DSB to either *white*+ (red eyes) or *white*Δ*9*+ (orange eyes) indicates interhomolog HR repair, as the only viable donor template was located on the homolog.

When using the *iwhite* donor template (Figure 2C), interhomolog recombination occurred at a frequency of 26.6 ± 1.5%, comparable to the 28.3% frequency of intrachromsomal HR repair in the DR-*white* assay (*P* > 0.05, two-tailed unpaired Student’s t-test). A homology-dependent effect on interhomolog recombination frequency was also observed. Interhomolog recombination decreased significantly by about 45% when using the *iwhite*Δ*9* donor template (14.1 ± 0.8%; Figure 2C; *P* < 10^−10^, two-tailed unpaired Student’s *t*-test), similar to the 40% decrease observed in the DR-*white*Δ*9* assay (*P* > 0.05, two-tailed unpaired Student’s t-test). Thus, interhomolog recombination in *Drosophila* occurs as frequently as intrachromsomal HR repair if no other donor template is available, and the efficiency of interhomolog repair is significantly affected by sequence divergence.

### Intrachromosomal donor templates are highly preferred in the premeiotic male germline

**Figure 3:**
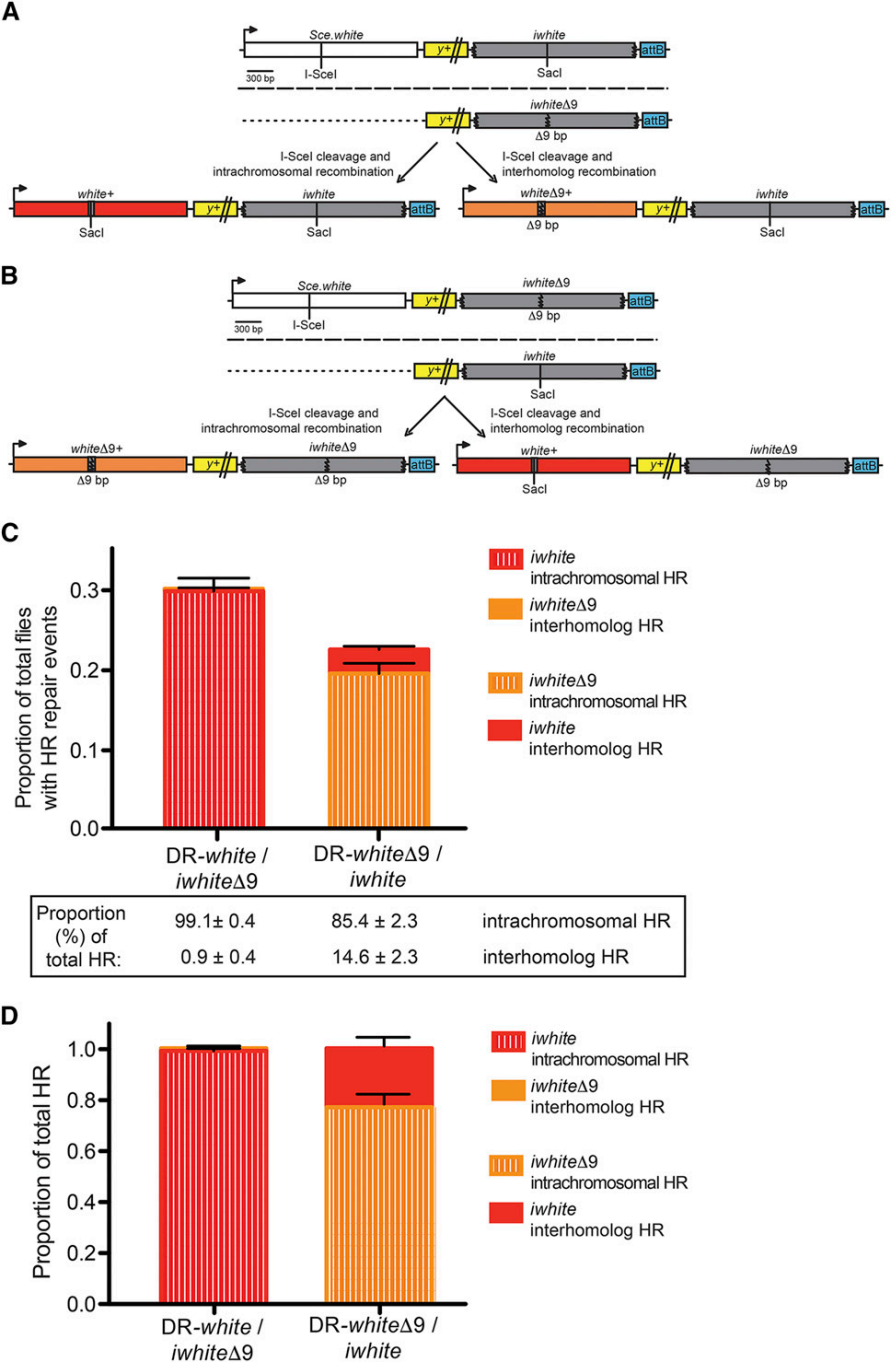
Intrachromosomal HR repair is preferred over interhomolog HR repair in mitotically dividing cells. (A) The intrachromosomal/interhomolog choice repair assay. Females carrying DR-*white* are crossed with males carrying the *iwhite*Δ*9* homolog donor template. HR repair of an I-*Sce*I induced DSB from the intrachromosomal *iwhite* donor template results in gene conversion of the *Sce.white* sequence to *white*+ (red box), resulting in red-eyed progeny. HR repair from the interhomolog *iwhite*Δ*9* donor template results in gene conversion of the *Sce.white* sequence to *white*Δ*9*+ (orange box), resulting in orange-eyed progeny. Homologs are separated by long dashed line; dotted lines are used for direct comparison with other figures. (B) The same intrachromosomal/interhomolog choice repair assay is performed with flies carrying DR-*white*Δ*9* and an *iwhite* homolog donor template. Intrachromosomal HR repair results in gene conversion of the *Sce.white* sequence to *whiteΔ9*+ (orange box), while interhomolog HR repair results in gene conversion to *white*+ (red box). (C) F2 progeny of 79 individual male germlines of DR-*white/iwhite*Δ*9* and 63 individual male germlines of DR-*white*Δ*9/iwhite* were scored. Average HR frequency out of total flies scored is shown. Error bars are S.E.M. Values given below are proportion of intrachromosomal or interhomolog HR out of total HR events ± SEM (D) Proportion of intrachromosomal (striped bars) or interhomolog (solid bars) HR out of total HR repair events in either DR-*white/iwhite*Δ*9* or DR-*white*Δ*9/iwhite* whole adult flies using TIDE analyses. Error bars are S.E.M.

Having established that interhomolog HR repair is possible and frequent in *Drosophila*, we investigated the relative contribution of intrachromosomal and interhomolog HR repair when donor templates are simultaneously available on both homologous chromosomes. Females carrying either DR-*white* or DR-*white*Δ*9* were crossed with males carrying the *iwhite*Δ*9* or *iwhite* interhomolog donor templates, respectively (Figure 3A and 3B).

When the *iwhite*Δ*9* sequence is used as the interhomolog donor template (Figure 3A), the frequency of interhomolog HR repair is only 0.3 ± 0.1% (Figure 3C). In contrast, when the *iwhite* sequence is used as the intrachromosomal donor template, the frequency of intrachromosomal HR repair is 29.4 ± 1.6%, accounting for the majority of all HR repair events (99.1 ± 0.4%, Figure 3C). Similarly, when *iwhite* is used as the interhomolog donor template (Figure 3B), the frequency of interhomolog HR repair is only 3.0 ± 0.4% (Figure 3C). When the more diverged *iwhite*Δ*9* sequence is used as the intrachromosomal donor template, the frequency of intrachromosomal HR repair is decreased (19.1 ± 1.3%) compared to intrachromosomal repair using the less diverged *iwhite* donor sequence. Although total HR frequency declined by about 25% (*P* < 0.001, two-tailed unpaired Student’s *t*-test), intrachromosomal recombination still occurred an impressive 85.4 ± 2.3% relative to total HR frequency (Figure 3C). Thus, despite the effects of sequence divergence on recombination frequency, the data strongly support a preference for intrachromosomal over interhomolog donor templates in the *Drosophila* male premeiotic germline.

### Intrachromosomal donor templates are highly preferred in non-germline tissues

The significant preference for intrachromosomal HR repair in the male premeiotic germline prompted us to investigate the HR repair preferences in other *Drosophila* tissues, including mitotically dividing somatic cells. As described above, heat-shock-induced DSBs were generated at the I-*Sce*I recognition site of *Sce.white* (Figure 3A and 3B). Cells in F1 larvae that contained repair events developed into adult tissues and were analyzed molecularly as a population of events. To quantify the proportion of intrachromosomal and interhomolog HR repair events across all tissues, DNA sequences from individual male and female F1 adults were analyzed with the established TIDE (Tracking across Indels by DEcomposition) algorithm (Janssen *et al.* 2016)(Supplemental File S1). The TIDE algorithm calculates the relative proportion of NHEJ with processing and HR repair events by comparing sequence deviations (Supplemental Files S2-S24) relative to the original I-*Sce*I recognition sequence in *Sce.white* (Supplemental Files S25-S26).

Of the TIDE-detectable repair events, NHEJ with processing ranged from 15.2 to 93.4% and total HR events were lower on average (ranging from 6.6 to 84.8%; Supplemental Table S1). Within the HR events, with an *iwhite* intrachromosomal donor template, the proportion of intrachromosomal HR repair events relative to total HR frequency was 98.9 ± 0.9% (Figure 3D, Supplemental Table S1). Notably, the proportion of intrachromosomal HR repair events was similar to that previously observed in the male premeiotic germline (99.1 ± 0.4%, *P* > 0.05, two-tailed unpaired Student’s *t*-test). Expectedly, with an *iwhite*Δ*9* intrachromosomal donor template, the proportion of intrachromosomal HR repair in all tissues decreased (76.9 ± 3.1%; Figure 3D, Supplemental Table S1). This observed decrease was similar to the decreased proportion of HR repair events with an *iwhite*Δ*9* intrachromosomal donor template in the male premeiotic germline (85.4 ± 2.3%, *P* = 0.03, two-tailed unpaired Student’s *t*-test). Furthermore, males and females showed no statistical difference in the relative proportion of intrachromosomal or interhomolog HR repair events (Supplemental Table S1, *P* > 0.05, two-tailed Student’s *t*-test). Thus, both somatic and premeiotic germline tissues demonstrate a homology-dependent preference for intrachromosomal HR repair.

## Discussion

Efficient repair of DSBs is critical for maintaining genomic integrity throughout a cell’s life cycle. While homologous recombination allows for accurate repair of DSBs, the factors involved in locating and discriminating between homologous donor templates remain unclear. Previous studies in *Drosophila* have shown that interhomolog recombination is possible (Engels *et al.* 1994; Rong and Golic 2003) but have not investigated whether there is a preference between allelic donor templates. Our results confirm that interhomolog recombination is possible and as frequent as intrachromosomal HR if no other sequence is available. However, given a choice between two allelic donor templates, the HR repair machinery exhibits a strong preference for intrachromosomal donor templates, which may be found elsewhere on the same chromosome (*i.e.*, in the DR-*white* assay or in repetitive DNA sequence) or on the sister chromatid. Interestingly, the chromosomal preference holds even when a less diverged donor template is available on the homolog, though total recombination frequencies do decrease. We propose that, in the presence of a diverged intrachromosomal template, repair shifts to true intersister HR (*e.g.*, repair from the identical *Sce.white* sequence on the sister chromatid), which cannot be distinguished phenotypically or molecularly in our assays from a no DSB or precise NHEJ event. These findings align with previous work in other eukaryotic systems that have established higher frequencies of recombination between sequences on sister chromatids (Kadyk and Hartwell 1992; Liang *et al.* 1998; Johnson and Jasin 2000; Engels *et al.* 1994).

Several models have been proposed for how the homology search is undertaken. One theory (the “null model”) suggests random sampling of DNA sequences to locate a homologous donor template (Barzel and Kupiec 2008). Given the extensive size of the eukaryotic genome and the relatively rapid kinetics of HR repair, the null model is unlikely (Barzel and Kupiec 2008). Our findings also suggest a more refined search process. Under a truly random homology search, one would not expect such a marked preference for intrachromosomal donor templates (>99% at times), especially when a less diverged donor template can be found on the homolog. The more prevailing theory is that preferences for intrachromosomal donor templates arise from the close proximity of intrachromosomal donor templates and sister chromatids (Johnson and Jasin 2000; Rong and Golic 2003; Barzel and Kupiec 2008).

The proximity theory, however, fails to explain the efficient use of interhomolog donor templates in *Drosophila* and ectopic sequences in yeast when no intrachromosomal donor template is available (Engels *et al.* 1994; Aylon and Kupiec 2003). The fact that interhomolog and ectopic recombination is even possible in these organisms implies that the HR repair machinery can effectively sample a wider share of the genome than previously suggested. One potential explanation for these differences is that homologous sequences in these organisms are paired throughout the cell cycle. Somatic pairing of homologous chromosomes in *Drosophila* has been long established (Metz 1916; McKee 2004). In yeast, chromosomes in interphase are arranged in specific configurations (known as Rabl or bouquet) so that allelic loci are relatively equidistant from their respective centromeric or telomeric regions (Barzel and Kupiec 2008). These global alignments of homologous sequences could explain the surprisingly efficient use of interhomolog HR in these organisms.

It is likely that proximity alone does not determine the choice of donor template. In fact, in accordance with previous work (Do *et al.* 2014), we consistently found a clear reduction in frequency of HR repair between slightly diverged sequences. Thus, the degree of homology between the broken and donor template remains a crucial aspect of HR repair. Interestingly, though intrachromosomal HR was preferred regardless of the identity of the intrachromosomal donor template, interhomolog HR was more pronounced when a less diverged sequence was located on the homolog. This finding suggests that the proximal donor template was rejected in favor of the allelic homologous sequence, despite being theoretically further away. A more intricate search method is therefore likely to be employed that considers both distance and homology requirements.

Recent research using single-molecule imaging has revealed a significant amount about the biochemistry of the RecA protein, a necessary component of the prokaryotic HR repair machinery. RecA, and its eukaryotic homolog Rad51 (*Drosophila DmRad51/spn-A)*, binds extensively to the resected 3′ ssDNA overhangs to create RecA-ssDNA filaments, which can extend many kilobases (reviewed in Bell and Kowalczykowski 2016). Furthermore, the search process, taking up to 50 min in *E. coli*, is limited to a small volume of the cell (Bell and Kowalczykowski 2016). These studies suggest the intriguing possibility that the search machinery is able to sample multiple sequences at once along the length of the resected filament. This could explain the slightly higher proportion of interhomolog HR when the less diverged donor template is on the homolog. Rapidly-sequential sampling of both allelic sites could allow the HR repair machinery to sporadically choose the less diverged template despite the presence of a proximal donor template in *cis*. Indeed, when the intrachromosomal donor template is less diverged, interhomolog HR occurs <1% of the time.

These results highlight a broader implication in genome editing. As Cas9-mediated genome editing technology becomes more widespread, the exact mechanisms underlying endonuclease-mediated DSB repair will need to be explained to ensure efficient and predictable repair outcomes (reviewed in Doudna and Charpentier 2014). In particular, researchers hoping to reverse pathogenic mutations via CRISPR/Cas9 using homology directed repair from ectopic sequences will need to consider both the necessary homology requirements as well as the potential for chromosomal rearrangements. A recent study in *Drosophila* demonstrated that up to 39% of Cas9-mediated DSBs result in recombination between homologous chromosome arms (Brunner *et al.* 2019). Thus, the possibility of unwanted repair events in diploid organisms cannot be ignored. Our own findings indicate that, despite stringent homology requirements and the possibility of interhomolog recombination, HR occurs preferentially between sequences located on the same chromosome, potentially reducing the efficiency of ectopic genetic transformations.
